# Profiling of *Burkholderia cepacia* Secretome at Mid-Logarithmic and Early-Stationary Phases of Growth

**DOI:** 10.1371/journal.pone.0026518

**Published:** 2011-10-26

**Authors:** Vanitha Mariappan, Kumutha Malar Vellasamy, Onn Haji Hashim, Jamuna Vadivelu

**Affiliations:** 1 Department of Medical Microbiology, Faculty of Medicine, University of Malaya, Kuala Lumpur, Malaysia; 2 Department of Molecular Medicine, Faculty of Medicine, University of Malaya, Kuala Lumpur, Malaysia; 3 University of Malaya Centre for Proteomics Research (UMCPR), University of Malaya, Kuala Lumpur, Malaysia; Institute of Microbial Technology, India

## Abstract

**Background:**

*Burkholderia cepacia* is a Gram-negative pathogen that causes serious respiratory infections in immunocompromised patients and individuals with cystic fibrosis. This bacterium is known to release extracellular proteins that may be involved in virulence.

**Methodology/Principal Findings:**

In the present study, *B. cepacia* grown to mid-logarithmic and early-stationary phases were investigated on their ability to invade and survive intracellularly in A549 lung epithelial cells in order to discern the fate of these bacteria in the pathogenesis of *B. cepacia* lung infections in *in vitro* condition. The early-stationary phase *B. cepacia* was demonstrated to be more invasive than mid-logarithmic phase. In addition, culture supernatants of *B. cepacia* obtained from these phases of growth were also demonstrated to cause different cytotoxic potency on the A549 human lung epithelial cells. Profiling of the supernatants using the gel-based proteomics approach identified 43 proteins that were commonly released in both the growth phases and 40 proteins newly-released at the early-stationary phase. The latter proteins may account for the higher cytotoxic activity of the early-stationary culture supernatant compared to that obtained at the mid-logarithmic phase. Among the newly-released proteins in the early-stationary phase supernatant were flagellar hook-associated domain protein (FliD), flagellar hook-associated protein (FlgK), TonB-dependent siderophore (Fiu), Elongation factor G (FusA), phosphoglycerate kinase (Pgk) and sulfatase (AslA) which are known for their virulence.

**Conclusion/Significance:**

Differences in the ability of *B. cepacia* to invade and survive intracellularly inside the epithelial cells at different phases of growth may improve our understanding of the varied disease progressions associated with *B. cepacia* infections. In addition, the identified culture supernatant proteins may be used as targets for the development of new strategies to control *B. cepacia* infection using agents that can block their release.

## Introduction

*Burkholderia cepacia* complex consists of nine closely related bacterial species (genomovars) that share some phenotypic or genotypic similarities. This organism is an opportunistic human pathogen associated with life-threatening pulmonary infections that affect immunocompromised individuals particularly patients with cystic fibrosis (CF) [1] and chronic granulomatous disease [2]. Furthermore, the bacteria have a potential for patient-to-patient spread and present as a threat for nosocomial acquired infection [1]. *B. cepacia* is known to adhere, colonise, invade, replicate, survive and persist in the host cells, as well as evade the host immune response [3]. Several researchers have demonstrated that the bacterial secretory products may play roles in colonisation or invasion by damaging the epithelial cells [4], [5]. In addition, *B. cepacia* is also known to produce several secreted products such as proteases [6], lipases [4], cytotoxins [7] and haemolysins [8] that are known to exhibit virulence [9] and contribute to severe inflammatory response that may induce host cell death [10].

*B. cepacia* secretory proteins play an important role in the pathogenesis of many infections. They are exposed to the host immune system and therefore may represent the inter-phase of bacterium-host interaction [11]. *In vitro* cultivation of bacteria results in the accumulation of a complex set of excreted proteins, collectively termed as the secretory or released proteins into the extracellular milieu. Many, but not all the secretory proteins are key virulence factors required for pathogenesis of infection [1]. Secretory proteins that cause virulence target host cellular processes including those linked to innate responses, and to disruption or alteration of pathways [12].

The number and types of proteins released into the culture supernatant have been demonstrated to be highly dependent on the cultivation properties and the duration of growth of the bacteria [13]. Numerous secretory proteins produced by other intracellular pathogens such as *Mycobacterium tuberculosis* [14], *Listeria monocytogens* [15] and *Helicobacter pylori* [16] have been previously identified by 2-DE and MS analyses. However, there are many other secretory proteins whose identities, structures and putative roles that have yet to be elucidated.

Thus, the objective of the present study was to determine the cytotoxic effect of *B. cepacia* supernatants collected at the mid-logarithmic and the early-stationary growth phases and to profile their protein compositions using the 2-DE-based proteomics approach. It was aimed to identify proteins that may be involved in pathogenesis of the disease.

## Methods

### Ethics statement

Ethics approval is not required in this study. No consent was required since no human participant was involved. The *B. cepacia* strain used in this study is a clinical isolate which was obtained from the old archive bacterial collection. Since our institute is a teaching hospital, bacterial isolates obtained as a part of diagnostic screening are archived and such cases are exempted from obtaining clearances. The study however has an Institutional Biosafety Committee approval.

### Bacterial strain, growth and culture conditions

The bacterial culture of *B. cepacia* CQK, isolated from a non-CF patient at the University Malaya Medical Centre, Malaysia, was prepared according to protocols earlier described [17]. Fifty µl of the bacterial culture was inoculated into 13 flasks, each containing 50 ml of fresh LB medium (representing 0 hour to 24 hours at 2-hourly intervals) and the cultures were grown at 37°C. Bacterial density at OD_600 nm_ and viable counts [18] were determined for the respective flasks. Three independent experiments were conducted for each supernatant sample. Culture sample was centrifuged at 20,000 *g* for 40 minutes at 4°C, after which the supernatant was harvested and filtered through a 0.22 µm pore size membrane filter to remove residual bacteria [17] The culture supernatant was concentrated 50-fold using ultrafiltration employing 10 kDa centricon ultra-free centrifugal filter units (Millipore, Massachusetts, USA). An isocitrate dehydrogenase assay to specifically detect bacterial lysis was performed according to Anderson *et al.* [13].

### Invasion assay

Approximately 5×10^5^ A549 cells were seeded into each well of a 24-well tissue culture plate containing 1 ml of RPMI growth medium containing 10% foetal calf serum and incubated at 37°C overnight. When 70–80% confluent monolayer was achieved, the RPMI growth medium was replaced with RPMI maintenance medium. The invasion assay was performed as described by Martin and Mohr (2000) [3] with slight modifications. The A549 cells were infected with *B. cepacia* grown to mid-logarithmic and early-stationary phase. The bacterial culture was centrifuged at 300 *g* for 5 minutes and the resulting pellet was incubated at 37°C in 1 ml RPMI for 30 minutes. The amount of bacterial inoculum was standardised to ∼1×10^8^ cfu/ml. Confluent monolayers of A549 cells were infected with bacterial inoculum at multiplicity of infection (MOI) of 1∶50. The plates were incubated for 3 hours respectively, at 37°C in 5% CO_2_ to allow bacterial adherence and invasion. After incubation, the monolayers were washed using 100 mM PBS (pH 7.0), after which 3 ml of RMPI containing a combination of ceftazidime (1 mg/ml) and gentamicin (1 mg/ml) was added into each well for 2 hours at 37°C to kill extracellular adherent bacteria. The cell monolayers were then washed three times with PBS and in addition, the final volume of PBS used to wash the monolayers was collected and plated on a NA to determine the number of live extracellular bacteria. The monolayer cells were then trypsined to detach the cells from the tissue culture wells and the detached cells lysed with 0.25% Triton X-100 prepared in PBS to quantitate the intracellular bacteria. Serial dilutions of the lysate was prepared and plated on NA to determine the bacterial counts [18]. This experiment was performed in triplicates and the results were averaged. A non-invasive strain of *Escherichia coli* strain, was used as a negative control

### Intracellular survival assay

The A549 epithelial cell monolayers were inoculated with *B. cepacia* bacteria harvested at mid-logarithmic and early-stationary phase at a MOI of 50∶1 for 3 hours. Following incubation at 37°C, the infected monolayers were washed with PBS and tissue culture medium containing a combination of ceftazidime (1 mg/ml) and gentamicin (1 mg/ml) was added and incubated for 2 hours to kill adherent extracellular bacteria. The medium was then replaced with antibiotic-free RPMI for 3 hours. The infected monolayers were trypsinised, lysed and the intracellular surviving bacteria were quantitated by serial dilution and plating over time as described above [18]. This experiment was repeated three times and the results were averaged.

### Cell viability assay

The culture supernatant harvested at every 2 hours interval was tested for cytotoxicity against A549 lung epithelial cells which were purchased from American Type Culture Collection (ATCC, Virginia, USA). The cells were maintained in RPMI 1640 medium (Sigma Chemical Co., St Louis, USA) supplemented with 2 mM L-glutamine and 10% FCS (Sigma Chemical Co., St Louis, USA) at 37°C with 5% CO_2_ atmosphere and saturating humidity. Cells were detached using trypsin (Sigma Chemical Co., St Louis, USA) and seeded at 2×10^4^ cells in each well of a 24-well cell culture plate, and incubated at 37°C overnight. The cells were then treated with 50 µl of an initial concentration equivalent to 50 µg/ml from each phase of growth and serially-diluted culture supernatants in RPMI medium. The treated cell lines were then incubated at 37°C overnight and the 3-(4,5-dimethylthiazol-2-yl)-2,5-diphenyltetrazolium bromide (MTT) assay to determine cell toxicity was performed according to the manufacturer's protocol (Sigma Chemical Co., St. Louis, USA). Briefly, MTT solution was added into each well, mixed thoroughly and incubated at 37°C for 4 hours. The media plus MTT solution was then removed and DMSO added into each well to dissolve the formation of formazan. The absorbance was measured using a microtitre plate reader (BioRad, Hercules, USA) at OD_570 nm_. The experiment was performed in triplicate and RPMI and LB media were used as controls.

### Two-dimensional gel electrophoresis (2-DE)

Fractions of culture supernatant of *B. cepacia* mid-logarithmic and early-stationary phases were subjected to precipitation using 25% (w/v) ice-cold trichloroacetic acid and prepared for 2-DE as previously described [17]. Protein concentrations were estimated using Bradford's method [19]. A total of 400 µg protein sample with the rehydration buffer (8 M urea, 2% CHAPS, 0.002% bromophenol blue) was applied onto the IPG strips (pH 4–7, 13 cm; GE Healthcare, Uppsala, Sweden). After 18 hours of rehydration under mineral oil, the protein was focused using IPGphor system (GE Healthcare, Uppsala, Sweden) as previously described [20]. The strips were then transferred onto 12% SDS-PAGE [15] for the second dimension electrophoresis. The resulting gels were stained using colloidal CBB [21]. Three biological growth experiments were performed to increase reproducibility of the results. The gels were scanned with an Image Scanner and analysed using the Image Master ™ 2D Platinum version 5.0 (GE Healthcare, Uppsala, Sweden).

### Mass spectrometry (MS) analysis

Selected protein spots were excised from the CBB G-250 stained 2-DE gels and digested with solution of sequencing-grade modified trypsin (Promega, Madison, USA). The peptides released from the gel were sent to the Australian Proteome Analysis Facility (APAF) for further analysis using matrix-assisted laser desorption/ionization – time of flight mass spectrometry (MALDI-TOF/MS).

### Protein identification and *in silico* analysis

Mass spectrometry data were submitted for database search using the MASCOT search engine (Matrix Science, London, UK). The following search settings were used: carboxymidomethylation of cysteine was the fixed modification; and oxidation of methionine was selected as the variable modification; maximum number of missed cleavages - 1; and peptide tolerance – 100 ppm [17]. All searches were performed against the non-redundant NCBI library (http://ncbi.nlm.nih.gov) database comprising annotated proteins of *B. cepacia* complex (Bcc). Functional class assignment of the identified proteins based on Cluster of Orthologous Groups (COG) of proteins functional categories (http://www.ncbi.nlm.nig.gov/COG/old/palox.cgi?fun=all). The bioinformatics database (www.cds.dtu.dk) was used to predict the mode of secretion (SignalP v3.0), cellular localisation (PSORT) and protein domains (TMHMM v2.0).

## Results

### Bacterial growth condition

**Figure 1:**
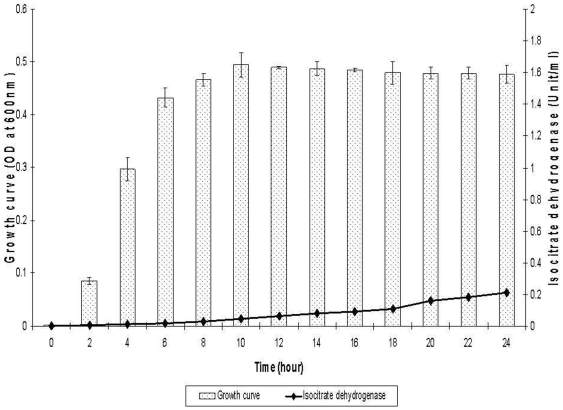
Growth curve of *Burkholderia cepacia* and isocitrate dehydrogenase activity at the different phases of growth. The culture was inoculated in LB medium and grown at 37°C from 0 h to 24 h. It was harvested at every 2 h intervals and the bacterial density was determined at OD_600 nm_. An isocitrate dehydrogenase assay to specifically detect bacterial lysis in the culture supernatant was preformed. These experiments were conducted in three independent replicates.

There was a good correlation observed between the optical density and viable counts of *B. cepacia* CQK culture at various phases of growth. The bacterial density increased rapidly from then on, reaching 1.07×10^7^ cfu/ml at 10 hours (early-stationary phase), with an OD_600 nm_ of 0.495, and 3.92×10^9^ cfu/ml after 24 hours, with an OD_600 nm_ of 0.477 (Fig. 1). The levels of isocitrate dehydrogenase, an intracellular enzyme activity which is used to reflect cell lysis, were found to be insignificant during the mid-logarithmic and early-stationary phases of bacterial growth.

### Invasion of A549 epithelial cells by *B. cepacia*

The ability of *B. cepacia* to invade and survive intracellularly in the epithelial cell was measured and compared between two different phases of growth (mid-logarithmic and early-stationary phase) (Table 1). The infected cells were incubated at time intervals of 3 hours to investigate the capacity of the bacteria to invade the epithelial cells. The *E. coli* that was used as a negative control did not demonstrate capability to invade into the epithelial cells. In addition, appropriate measures were taken to ensure that no extracellular bacteria were present before the cells were lysed.

**Table 1:** Mean percentage of invasion (%) with standard deviation MOI 1∶10 and1∶50.

*B. cepacia* growth phase	Invasion	
	MOI 1∶10	MOI 1∶50
Mid logarithmic	0.0044±0.0016	0.0089±0.0016
Early-stationary	0.0088±0.0032	0.0155±0.0016

At MOI 1∶10, three hours of post-infection, the number of bacterial cells penetrated into A549 cells were low, with 0.0044±0.00016% (mid-logarithmic phase) and 0.0088±0.00032% (early-stationary phase). The invasion capacity at an MOI 1∶50 was comparatively higher than at MOI 1∶10 at the different phases of growth. The mean percentage of invasion increased approximately two-fold at MOI 1∶50 post-infection to 0.0089±0.0016% (mid-logarithmic phase) and 0.0155±0.0016% (early-stationary phase). The percentage of invasion was found to be dependent upon the inoculum size used. The early-stationary phase bacteria were more invasive at both the MOI of 1∶10 and 1∶50 than the mid-logarithmic phase. The isolate showed a variable degree of invasion capacity at different bacterial phases of growth with different MOI.

### Intracellular survival of *B. cepacia*

**Figure 2:**
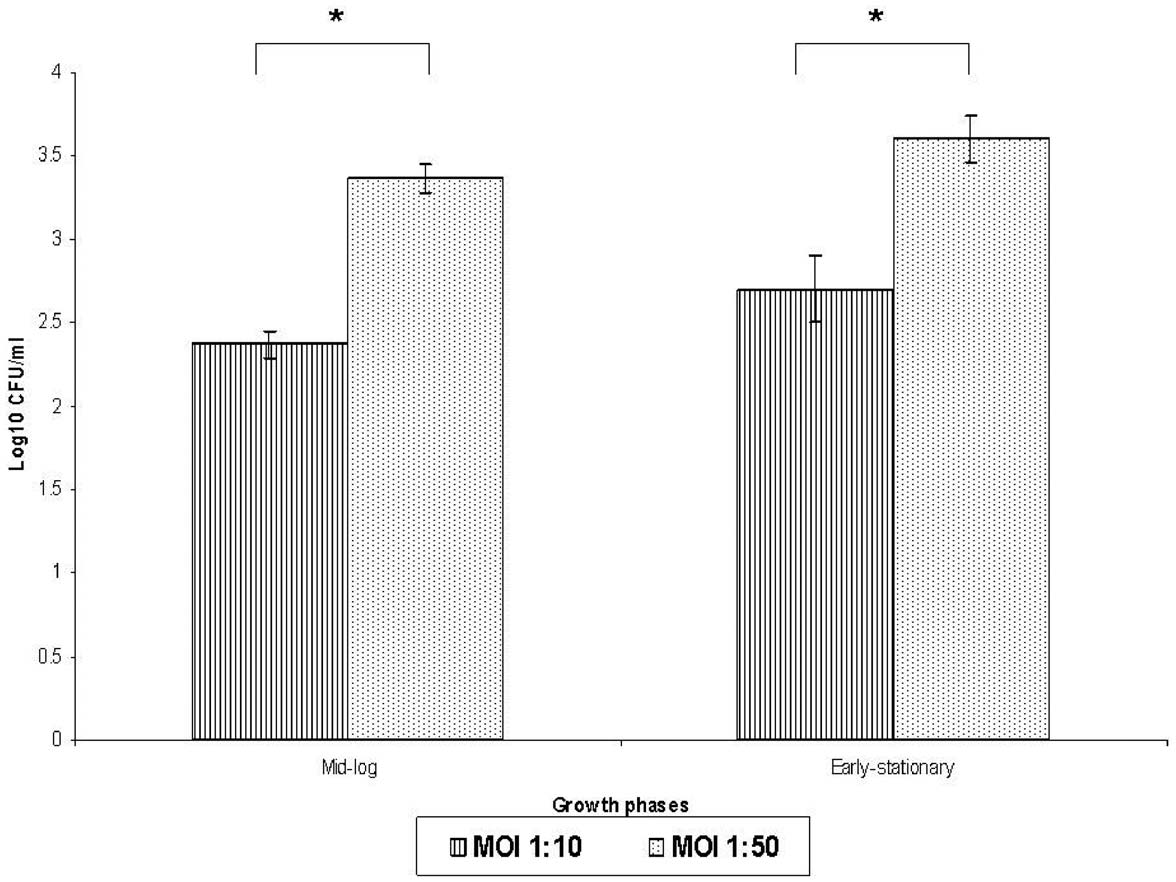
The intracellular survival assay of *B. cepacia* (mid-logarithmic and early-stationary phases of growth) after 3 hours of post-infection with MOI of 1∶10 and 1∶50 was as Log_10_ cfu/ml of the bacteria recovered. These experiments were conducted in three independent replicates. The error bars indicate the standard deviation. The significance has been indicated using *.

The ability of the *B. cepacia* isolates to enter cultured human epithelial cells with different invasion frequencies prompted the examination of intracellular replication rate. The inoculum size and growth phases used in intracellular survival assay were identical to invasion assay. The comparison of intracellular survival capacity at different phases of culture growth (mid-logarithmic, and early-stationary phase) was indicated as log_10_ (Fig. 2). The intracellular survival assays at 3 hours with MOI 1∶10 demonstrated that the number of *B. cepacia* survived intracellularly increased slightly from log_10_ 2.37 to log_10_ 2.70 at mid-logarithmic and early-stationary phase, respectively. However, with MOI 1∶50 intracellular replication, slow replication of *B. cepacia* in the epithelial cells was demonstrated, where the number of bacteria was observed to increase by log_10_ 0.24. Overall, the *B. cepacia* strain was shown to be able to survive and replicate slowly intracellularly, however it depends on the number of bacteria that invaded the A549 cells.

### Effect of bacterial culture supernatant on A549 cells

**Figure 3:**
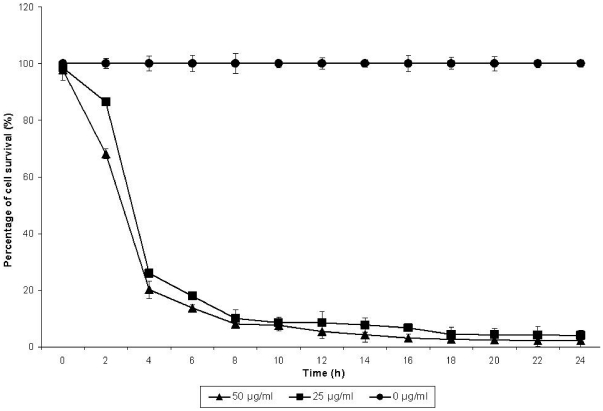
Effect of *Burkholderia cepacia* culture supernatant on A549, human lung epithelial cells. The cytotoxicity effect was determined using MTT assay. The experiment was performed in triplicates with RPMI and LB media used as control. The error bars indicate the standard deviation. The error bars indicate the standard deviation.

The cytotoxic effect of the bacterial culture supernatants, harvested at different time-points in the growth cycle of the bacteria was demonstrated when the supernatants were added to cultures of A549 cells (Fig. 3). Exposure of the A549 cells to the culture supernatants obtained at 2 hours, with protein concentrations of 25 and 50 µg/ml, caused 14–32% cell death. Survival was further reduced by 80–96% when 4 hours to 14 hours culture supernatants were added to the A549 cell cultures. Only 2–6% of the A549 cells were found to survive in the presence of 16 hours and 24 hours culture supernatants of the bacteria.

### Profiling of culture supernatant proteins

**Figure 4:**
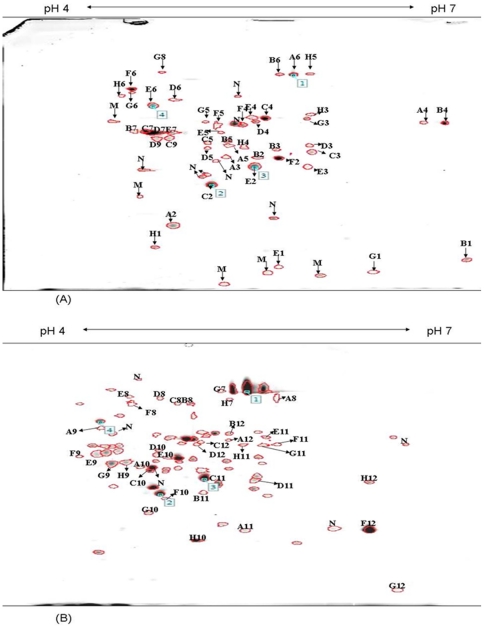
Analysis of *B. cepacia* culture supernatant by 2-DE. The supernatant of *B. cepacia* grown to mid-logarithmic (panel A) and early-stationary (panel B) phases in LB medium were prepared using TCA precipitation method and analysed using 2-DE. A total of 400 µg of culture supernatant was separated on an IPG strip pH 4–7 in the first dimension, followed by the separation on SDS-12% PAGE for the second-dimension separation. The separated proteins were detected by CBB G-250 staining. Marked spots indicate proteins that were identified. N refers to spots that were not possibly identified by MALDI-TOF.

Figure 4 demonstrates the 2-DE protein profiles of *B. cepacia* supernatants harvested from cultures grown at mid-logarithmic (panel A) and early-stationary (panel B) phases. While 56 protein spots were detected in the supernatant of cultures collected at the mid-logarithmic phase, 83 spots were resolved from the early-stationary phase culture supernatant. Among the protein spots detected in the supernatant of cultures collected at the mid-logarithmic phase, 13 were apparently not present in the early-stationary phase culture supernatant. On the other hand, 40 newly-released spots were detected in the supernatant obtained from the early-stationary phase bacterial cultures.

### Identification of culture supernatant proteins

Out of a total of 96 protein spots detected in the 2-DE profiles, 81 were subsequently identified using MS and database search (Table S1). The remaining 15 protein spots were of low abundance and in insufficient quantities for the MALDI-TOF analysis. Upon identification, several of the protein spots turned up to be isoforms of the same proteins. For example, DnsK and FliD were apparently resolved into three different isoforms in the 2-DE gels, while two isoforms were detected for Eno, AlaDH, TktA, Fiu FusA and hypothetical protein.

### *In silico* analysis of bacterial released proteins

When analysed using bioinformatics software, three of the identified proteins that were exclusively detected in the supernatant of bacterial culture obtained at the mid-logarithmic phase were found to be involved in metabolism (i.e., purA, GuaB and TpiA), while the other three were associated with cellular processes (i.e., DnaK (3 isoforms), RfaG and GlmU; Table S2). Among proteins that were commonly detected in the mid-logarithmic and early-stationary phase culture supernatants, 21 were apparently involved in metabolism, 12 with cellular processes and six in information storage and processing (Fig. S1, panel A). One of the proteins (i.e., AdhP) which were commonly detected in the mid-logarithmic and early-stationary phase culture supernatants could not be characterised. Among proteins detected in the supernatants collected at mid-logarithmic phase culture, 54.3% were found to be located in the cytoplasm, 8.7% in the cytoplasmic membrane and 32.6% had no known location (Fig. S1, panel B). Three proteins, i.e., MetK, Spr and FtsZ, were apparently associated with multiple locations. FliD was predicted as an extracellular protein and Fiu as an outer membrane protein. Among the 35 proteins detected as newly-released at the early-stationary phase of bacterial growth, 12 were involved in metabolism, 10 in cellular processes, eight in storage and processing of information and five were not characterised (Fig. S2, panel A). Among the proteins released at the early-stationary phase, 40% were predicted to be located at the cytoplasm and another 40% had no known location; 5.7% in the cytoplasmic membrane; 5.7% in the outer membrane; and 8.6% were predicted to be the extracellular secreted proteins (Fig. S2, panel B). When SignalP v3.0 was used to predict presence of cleavage sites for bacterial signal peptidases, five proteins commonly expressed in the supernatants at the mid-logarithmic and stationary phase cultures (i.e., FabB, PlsC, Spr, FliD, and Fiu) and four proteins that were newly-released detected at the early-stationary phase of growth (i.e., BaeS, FlgK, AslA and three isoforms of FliD) were predicted as secretory proteins of the classical Sec pathway. The cell motility and secretion proteins, FliD and FlgK, were predicted as secretory proteins, as expected. The TMHMM v2.0 algorithm predicted the mid-logarithmic phase proteins, PlsC and Spr, as integral membrane-associated proteins with one helix. In addition, two newly-released proteins detected in the early-stationary phase bacterial culture supernatant, i.e., BaeS and AslA, were also identified as integral membrane-associated proteins, with two helices and one helix, respectively.

## Discussion

*B. cepacia* is known to adhere, invade, replicate, survive and persist in the host cells, as well as evade the host immune response [3]. Many studies have demonstrated the ability of *B. cepacia* to invade and survive within epithelial cells *in vitro* [3], [22]–[24]. In addition, proteins secreted by this pathogen are of special interest because they come in direct contact with the host tissues and may mediate pathogenesis. Several studies have identified proteins secreted by *B. cepacia* as the virulence factors involved in pathogenesis [25], [26]. The secretory proteins have also been suggested as potential diagnostic biomarkers and/or candidates for development of vaccine and therapeutic intervention [27].

In this study, *B.* cepacia harvested at mid-logarithmic and early-stationary phases were tested for their ability to invade and multiply inside epithelial cells within 3 hours of exposure. During the mid-logarithmic phase, the bacterial cells are dividing actively and the number of bacteria increased as the duration of growth increased. In the early-stationary phase, the bacteria were still dividing, but very slowly perhaps due to low carbon reserves in the medium. The results suggest that the invasion efficiency of *B. cepacia* was directly related to the phases of growth at which the bacterial cells were harvested and used for the invasion studies. The bacterial invasiveness at the early-stationary phase of growth was greater than the mid- log phase in this *in vitro* condition.

Similarly, the increased number of bacteria inoculated also resulted in the increase of invasive ability. Collectively, these factors may contribute to the virulence potential of pathogenic *B. cepacia*. The possible explanation of this could be due to the secretion of different type of bacterial proteins or enzymes which are known to play roles in colonisation or invasion by damaging the epithelial cells. In addition, the secretory enzymes are also known to damage the host cells and/or have direct effect of facilitating the bacterial growth and cell to cell spreading. The damage to the host is part of the pathology of infectious disease as a result of bacterial invasive activity.

In addition, the supernatants of *B. cepacia* cultures obtained at different time-points of growth were initially demonstrated to exert cytotoxic effects on the mammalian A549 human lung epithelial cells. The cytotoxic effects apparently increased with time, suggesting that this might be due to the increasing amounts and/or types of cytotoxic proteins that accumulated in the culture supernatant during the growth cycle [28]. The variation in cytotoxic potency of supernatants collected at the different growth phases, particularly the mid-logarithmic and early-stationary phases, suggests differences in the pathogenic potentials of *B. cepacia* to infect and cause disease in man.

In an attempt to identify and predict the roles of the proteins in the supernatants during the mid-logarithmic and early-stationary phases of *B. cepacia* culture, a gel-based proteomics study was performed. When separated by 2-DE at pH 4–7, 37 proteins were identified and commonly mapped at both the mid-logarithmic and early-stationary culture phases of the *B. cepacia* secretome. Aside from these, 13 proteins were exclusively identified in the mid-logarithmic phase and 40 proteins were newly-released in the early-stationary phase. This was generally in slight variation with our previous report [17] and that of others [27], [29], but the differences may be attributed to the variations in the time of collection of the supernatants as well as the inherent genetic differences in the strains used in the experiments [17]. When taken together with the findings of the cytotoxicity experiments, the differential protein profiles detected in the mid-logarithmic and early-stationary growth phases of the *B. cepacia* secretome suggests that the proteins may have contributed to the different cytotoxic effects of the supernatants.

The *in silico* technologies such as PSORT and SignalP facilitated prediction of cellular localisation and signal peptide of the *B. cepacia* released proteins. Of the 46 identified protein spots released at the mid-logarithmic phase, 50% were predicted to have originated from the cytoplasm. This is similar to the reported secretome proteins of *B. cenocepacia* H111 [29]. Analysis of the 46 identified proteins detected at the mid-logarithmic phase by COG demonstrated that 52% were involved in metabolism, which is similar to that reported in *Shigella flexneri* [30]. This was not surprising as bacteria are known to actively multiply at this phase of growth to increase its population number for host colonisation. Many of the proteins synthesised during this phase would therefore be involved in the metabolism of nutrients to sustain growth as well as biogenesis of new cells. The active multiplication of bacterial cells during this phase was also reflected by detection of the cell division proteins, FtsZ and FtsA, in the supernatants collected, which explains the increased in the number of bacteria that invaded the host cells. Increase in both FtsZ and FtsA, which was required to cause early central division of the cells, was also earlier demonstrated in the *Escherichia coli* [31].

Among the six identified proteins that were exclusively released at the mid-logarithmic growth phase, three were metabolic proteins (i.e., purA, guaB and TpiA) while the remainder (i.e., RfaG, GlmU and DnaK) were involved in cellular processes. These proteins may not be required during the early-stationary phase, and thus, no longer secreted and degraded. DnaK, a molecular chaperone used in protein folding [32], was present in three isoforms in the *B. cepacia* mid-logarithmic phase secretome, and all three isoforms were absent at the early-stationary phase of growth. Presence of DnaK during the mid-logarithmic growth phase could be attributed to its role in protein folding during biogenesis of proteins in the dividing cells [32], [33]. Its absence at the early-stationary phase was most likely due to the lack of biogenesis in cells that were not dividing. Unlike DnaK, the 60 kDa chaperonin, GroL, appeared to be released in both the mid-logarithmic and early-stationary phases of bacterial growth.

The data of our present study demonstrated a considerable number of proteins being continuously released by *B. cepacia* in both the mid-logarithmic and early-stationary phases of growth. This was most likely due to their constitutive secretion/release and/or stability of the proteins which did not undergo denaturation and many of these proteins were enzymes involved in metabolism. Some of the secreted proteins are effector molecules while others possess enzymatic activities for housekeeping purposes which may also be associated with pathogenicity [26].

At the early-stationary phase of the bacterial culture, 40 of the 83 protein spots that were identified appeared to be newly-released proteins. Six proteins, i.e., Eno, AlaDH, TktA, Fiu, FusA and FliD, were resolved into their isoform subunits. Presence of the isoforms was probably due to differences in post-translational modifications, although very little is known on the role of post-translational modifications in bacterial physiology [34]. The early-stationary phase of growth is a state of stability where the bacteria do not actively multiply. Therefore, the release of new proteins into the supernatant was more likely to be involved in the pathogenesis mechanism.

Aside from known secretory proteins, approximately 50% of the proteins released into the supernatant at the early-stationary phase of growth in this study were predicted to have originated from the cytoplasm. As previously observed, some cytoplasmic proteins may indeed be secreted into the environment during the different stages of the cell cycle [35]. Among the proteins that were identified at the early-stationary phase, 35% were found to be involved in metabolic functions. The release of the metabolic enzymes at this phase was possibly for energy conservation and bacterial survival due to depletion of the limited carbon source in the growth medium. On the other hand, metabolic proteins have also been suggested to play a role in virulence [34], and their release at the early-stationary phase may also be for the purpose of adherence and/or invasion of the host cells.

Two of the proteins that were newly-released into the supernatant during the early-stationary phase of the *B. cepacia* culture, FliD and FlgK, were presumably generated during the flagellum assembly or degradation. *B. cepacia* culture supernatant flagellins were also identified as potential diagnostic candidates [27]. Relatively high amounts of flagellins have been previously reported to be released by many *Burkholderia* species into their culture supernatants [27], [29], [34]. The secretion of flagellins improved motility, which is required for successful adherence and colonisation at this stage of the growth cycle and as such this finding is consistent with the increase of invasion efficiency observed in this present study. Both of these proteins had also been previously reported to be present in the *B. cepacia* early-stationary phase culture supernatant by others [17], [36].

Apart from the flagellins, FusA [33], Fiu [37], Pgk [38] AslA [39] GroL [40] and GapA [41] are also proteins known to be associated with virulence. The release of FliD, FlgK, FusA, Fiu, Pgk and AslA into the culture supernatant of *B. cepacia* at the early-stationary phase of growth provides some explanation for its higher cytotoxic activity on the A549 cells compared to the supernatant that was obtained at the mid-logarithmic phase. Differences in the ability of *B. cepacia* to invade and survive intracellularly inside the epithelial cells at different phases of growth may also improve our understanding of the varied disease progressions associated with *B. cepacia* infections. The virulent proteins that were identified in the present study may used as targets for the development of new strategies to control the *B. cepacia* infection using agents that can block their secretion.

## Supporting Information

Figure S1 Protein categories and the subcellular localisation of identified *Burkholderia cepacia* culture at the mid-logarithmic phase. Functional protein categories were predicted using COG, while subcellular localisation was predicted using PSORT. Panels A and B refer to protein functions and cellular location, respectively.
(TIF)

Figure S2 Protein categories and the subcellular localisation of identified *Burkholderia cepacia* culture at the early stationary phase. Functional protein categories and subcellular localisation were predicted using COG and PSORT, respectively. Panel A refer to protein functions and panel B refers to cellular location.
(TIF)

Table S1 Identification of *Burkholderia cepacia* culture supernatant proteins using MALDI-TOF analysis.
(DOC)

Table S2 *In silico* analysis of *Burkholderia cepacia* culture supernatant proteins.
(DOC)
