# Supplementary material for: Profiling of Burkholderia cepacia Secretome at Mid-Logarithmic and Early-Stationary Phases of Growth
Source: PLoS One. 2011 Oct 26;6(10):e26518. doi: 10.1371/journal.pone.0026518 (PMC3202529; doi:10.1371/journal.pone.0026518)
Supplement: Table S1 — Identification of Burkholderia cepacia culture supernatant proteins using MALDI-TOF analysis. (DOC) [file pone.0026518.s003.doc]

Table S1: Identification of *Burkholderia cepacia* culture supernatant proteins using MALDI-TOF analysis

| **Spot** | **Protein** | **Accession no ^a^** | **Sequence coverage (%)** | **No. of peptides matched** | **Theo/exp MW** | **Theo/exp pI** |
| --- | --- | --- | --- | --- | --- | --- |
| ***Exclusive to mid-log phase culture supernatant*** | | | | | | |
| B1 | Adenylosuccinate synthetase (purA) | YP_773629 | 9 | 5 | 48.13/47.09 | 6.04/5.26 |
| A4 | Inositol-5-monophosphate dehydrogenase (GuaB) | YP_001119735 | 26 | 13 | 51.99/52.55 | 6.91/6.17 |
| A2 | Triosephosphatase isomerase (TpiA) | YP_369817 | 58 | 17 | 26.10/26.47 | 5.59/4.94 |
| E1 | Glysosyl transferase (RfaG) | ZP_02907643 | 29 | 20 | 188.30/56.67 | 5.78/4.94 |
| H3 | UDP-N-acetylglucosamine pyrophosphorylase (GlmU) | YP_622240 | 20 | 10 | 48.00/49.73 | 5.66/5.58 |
| F6 | Molecular chaperone (DnaK) | YP_002099175 | 49 | 36 | 69.61/83.51 | 4.95/4.68 |
| G6 | Molecular chaperone (DnaK) | YP_002099175 | 49 | 35 | 69.61/80.70 | 4.95/4.68 |
| H6 | Molecular chaperone (DnaK) | YP_002099175 | 46 | 31 | 69.61/80.02 | 4.95/4.60 |
| ***Commonly detected in mid-log and early-stationary phase culture supernatant*** | | | | | | |
| B4 | Inositol-5-monophosphate dehydrogenase (GuaB) | YP_001119735 | 48 | 32 | 51.99/53.11 | 6.91/6.52 |
| A5 | Glu/Leu/Phe/Val dehydrogenase (GdhA) | YP_772456 | 42 | 22 | 46.48/40.05 | 6.28/5.13 |
| F2 | Glyceraldehyde-3-phosphate dehydrogenase (GapA) | YP_620043 | 44 | 13 | 36.23/35.93 | 6.21/5.37 |
| B7 | Phosphopyruvate hydratase (Eno) | YP_001120023 | 45 | 18 | 45.67/46.67 | 4.77/4.66 |
| C7 | Phosphopyruvate hydratase (Eno) | YP_001120023 | 58 | 26 | 45.67/46.50 | 4.77/4.85 |
| B2 | Propionyl-CoA carboxylase | YP_620646 | 45 | 15 | 57.45/35.21 | 6.62/4.75 |
| B5 | 3-oxoaclyl-(acyl carrier protein) synthase II (FabB) | YP_001118890 | 33 | 17 | 42.86/43.90 | 5.71/5.14 |
| H5 | β-ketoacyl synthase | YP_001580137 | 28 | 12 | 238.14/220.64 | 5.89/6.60 |
| A6 | β-ketoadipyl CoA thiolase | YP_001583337 | 25 | 10 | 168.72/41.67 | 5.66/5.02 |
| G2 | FAD dependent oxidoreductase (DadA) | YP_366428 | 43 | 4 | 41.95/9.34 | 6.35/6.34 |
| B3 | Aspartate-semialdehyde dehydrogenase (Asd) | YP_002099660 | 33 | 14 | 42.49/38.12 | 5.65/5.36 |
| D3 | Alanine dehydrogenase (AlaDH) | YP_775531 | 5 | 22 | 38.49/40.32 | 6.15/5.51 |
| H4 | Alanine dehydrogenase (AlaDH) | YP_775531 | 54 | 20 | 38.49/40.08 | 6.15/5.22 |
| D5 | Acetylornithine deacetylase (ArgE) | YP_001773821 | 40 | 17 | 44.40/39.80 | 5.29/5.10 |
| C3 | 3-methyl-2oxobutanoate dehydrogenase (AcoA) | YP_620645 | 46 | 23 | 44.84/41.42 | 6.08/5.52 |
| C4 | Glycine dehydrogenase | YP_001578333 | 61 | 29 | 59.39/104.20 | 5.40/5.40 |
| E4 | Branched chain α-keto acid dehydrogenase (AceF) | YP_620647 | 45 | 24 | 46.95/46.01 | 5.96/5.40 |
| F5 | Putative dihydrolipoamide dehydrogenase (Lpd) | YP_002231334 | 27 | 13 | 61.54/49.64 | 5.44/5.25 |
| E7 | Vanillate monooxgenase | P_001811788 | 19 | 8 | 51.38/39.28 | 5.60/4.87 |
| C9 | Phospholipid/glycerol acyltransferase | YP_621966 | 23 | 7 | 29.40/25.77 | 5.25/5.20 |
| E5 | S-adenosylmethionine synthetase (MetK) | YP_622339 | 34 | 16 | 42.71/43.47 | 5.04/5.14 |
| G3 | Cell-wall associated hydrolase (Spr) | YP_001579403 | 31 | 21 | 24.23/53.69 | 9.26/6.36 |
| G4 | D-Alanine-A-alanine ligase | YP_001118384 | 51 | 26 | 33.36/33.38 | 5.66/4.9 |
| D4 | Flagellar hook-associated 2 domain protein (FliD) | YP_00157842 | 55 | 22 | 49.52/51.51 | 4.94/5.36 |
| D6 | Sulfatase (AslA) | YP_625056 | 29 | 20 | 67.39/73.33 | 9.02/6.19 |
| C5 | Cell division protein (FtsZ) | YP_001118387 | 36 | 16 | 41.64/40.65 | 4.87/5.19 |
| G8 | Cell division protein (FtsA) | YP_367894 | 14 | 7 | 43.82/46.56 | 4.95/5.29 |
| H1 | Peroxiredoxin (AhpC) | YP_0015844661 | 29 | 7 | 20.74/20.86 | 5.08/4.76 |
| E3 | TonB-dependent siderophore (Fiu) | YP_001119430 | 19 | 10 | 83.10/43.06 | 8.49/6.85 |
| F4 | ATP-dependent protease ATP- binding | YP_001121001 | 40 | 21 | 50.00/50.09 | 5.58/5.25 |
| E6 | 60 kDa chaperonin (GroL) | YP_002232255 | 45 | 33 | 56.98/57.37 | 5.13/4.79 |
| B6 | Phage SP01 DNA polymerase | YP_001765345 | 32 | 10 | 30.76/35.93 | 5.17/4.66 |
| D7 | DEAD/DEAH box helicase | YP_001578819 | 68 | 35 | 49.75/51.74 | 5.70/4.66 |
| G1 | Ribosome recycling factor (Frr) | YP_001119760 | 44 | 12 | 20.83/20.67 | 7.88/5.79 |
| E2 | Elongation factor Ts (Tsf) | YP_001579443 | 66 | 24 | 31.22/32.56 | 5.52/5.33 |
| G5 | Asparty/glutamly-tRNA amidotransferase (GatB) | YP_370696 | 58 | 37 | 53.51/53.36 | 5.06/5.10 |
| D9 | Tyrosyl-tRNA synthase | YP_368014 | 27 | 12 | 47.50/45.53 | 5.90/4.66 |
| A3 | Zn-dependent alcohol dehydrogenase (AdhP) | YP_002094374 | 34 | 12 | 37.91/37.45 | 6.26/5.19 |
| ***Newly-released into early-stationary phase culture supernatant*** | | | | | | |
| F11 | Adenylosuccinate synthetase (PutA) | YP_001119567 | 47 | 26 | 48.17/47.82 | 6.42/5.43 |
| C8 | Pyruvate kinase (PykF) | YP_001811920 | 37 | 29 | 51.74/46.45 | 5.98/4.6 |
| F8 | Transketolase (TktA) | YP_625131 | 33 | 24 | 74.21/90.32 | 6.25/5.13 |
| F9 | Transketolase (TktA) | YP_625131 | 27 | 22 | 74.21/72.46 | 6.25/4.96 |
| E10 | Phosphoglycerate kinase (Pgk) | YP_370197 | 64 | 22 | 41.27/42.56 | 5.26/5.18 |
| G10 | 3-oxoacid CoA-transferase (AtoD) | YP_368850 | 46 | 12 | 22.50**/**25.10 | 5.26/6.35 |
| G12 | (3R)-hydroxymyristoyl-ACP dehydratase | YP_773931 | 62 | 10 | 18.02/16.07 | 8.67/6.18 |
| E9 | 2-aminoethylphosphonate-pyruvate transaminase | YP_370735 | 70 | 7 | 41.78**/**40.64 | 6.45/6.13 |
| A12 | Aspartate ammonia-lyase (AspA) | YP_624161 | 36 | 24 | 50.44/52.50 | 5.76/5.38 |
| A8 | Pyruvate carboxylase | YP_777635 | 35 | 23 | 77.62/127.77 | 5.10/5.4 |
| E11 | Aldehyde dehydrogenase | YP_622400 | 47 | 25 | 52.94/51.66 | 6.13/5.46 |
| B12 | Delta-1-pyrroline-5- carboxylate dehydrogenase | YP_001763430 | 51 | 27 | 140.15/56.42 | 5.94/5.43 |
| F12 | Glycosyl transferase (RfaG) | YP_772651 | 68 | 20 | 52.55**/**139.23 | 6.75/5.80 |
| H12 | Capsule polysaccharide biosysnthesis | YP_620181 | 44 | 20 | 44.87/34.46 | 8.83/5.85 |
| B8 | Integral membrane sensor signal histidine kinase (BaeS) | YP_001810823 | 43 | 39 | 47.89**/**50.66 | 7.83/7.22 |
| H9 | Flagellar hook-associated protein (FlgK) | YP_002101099 | 55 | 20 | 46.55/46.22 | 4.67/5.04 |
| H11 | Flagellar hook-associated 2 domain protein (FliD) | YP_001578342 | 54 | 29 | 49.52/48.12 | 4.94/5.52 |
| C12 | Flagellar hook-associated 2 domain protein (FliD) | YP_001578342 | 57 | 28 | 49.52/51.51 | 4.94/5.36 |
| D12 | Flagellar hook-associated 2 domain protein (FliD) | YP_001578342 | 59 | 27 | 49.52/50.76 | 4.94/5.20 |
| D10 | Sulfatase (AslA) | YP_625056 | 38 | 27 | 67.39/73.33 | 9.02/6.19 |
| C11 | TonB-dependent siderophore (Fiu) | YP_001119430 | 40 | 23 | 83.10/43.06 | 8.49/5.76 |
| D11 | TonB-dependent siderophore (Fiu) | YP_001119430 | 38 | 21 | 83.10/40.12 | 8.49/6.85 |
| D8 | Polyribonucleotide nucleotidyltransferase (Pnp) | YP_002097866 | 54 | 47 | 78.15/78.34 | 5.36/5.13 |
| E8 | Elongation factor G (FusA) | YP_001578438 | 53 | 41 | 77.49/76.53 | 5.34/4.91 |
| A9 | Elongation factor G (FusA) | YP_622633 | 45 | 19 | 77.51/43.03 | 5.35/4.82 |
| F10 | Methionine aminopeptidase (Map) | YP_001580338 | 50 | 17 | 29.36/27.22 | 5.93/5.13 |
| A11 | Peptidyl-tRNA hydrolase (Pth) | YP_622064 | 41 | 9 | 22.07/21.32 | 8.93/9.02 |
| G9 | DNA-directed RNA polymerase (RpoA) | YP_001578467 | 49 | 35 | 35.67/34.97 | 5.63/4.89 |
| B11 | ROK family protein (NagC) | YP_001579895 | 37 | 14 | 44.16/34.67 | 6.22/5.30 |
| H7 | Two-component transcriptional regulator | YP_001120590 | 22 | 9 | 18.26/26.03 | 6.40/5.30 |
| G7 | Hypothetical protein | YP_002096038 | 44 | 16 | 10.12/31.62 | 5.11/5.42 |
| A10 | Hypothetical protein | YP_366327 | 40 | 19 | 40.58**/**45.62 | 5.56/4.94 |
| C10 | Hypothetical protein | YP_366327 | 46 | 26 | 40.58**/**42.21 | 5.56/5.19 |
| H10 | Hypothetical protein | YP_002101101 | 75 | 13 | 36.00**/**35.88 | 8.29/11.15 |
| G11 | Conserved hypothetical protein (chitinase) | YP_002100441 | 46 | 20 | 50.33/50.09 | 5.72/6.14 |

^a^ Accession number obtained from GenBank Database
