# Supplementary material for: Profiling of Burkholderia cepacia Secretome at Mid-Logarithmic and Early-Stationary Phases of Growth
Source: PLoS One. 2011 Oct 26;6(10):e26518. doi: 10.1371/journal.pone.0026518 (PMC3202529; doi:10.1371/journal.pone.0026518)
Supplement: Table S2 — In silico analysis of Burkholderia cepacia culture supernatant proteins. (DOC) [file pone.0026518.s004.doc]

Table S2: *In silico* analysis of *Burkholderia cepacia* culture supernatant proteins

| **Spot** | **Protein functional category ^a^** | | **Protein** | **Subcellular location ^b^** |
| --- | --- | --- | --- | --- |
| ***Exclusive to mid-log phase culture supernatant*** | | | | |
| B1 | METABOLISM | ***Nucleotide transport and metabolism*** | Adenylosuccinate synthetase (purA) | unknown |
| A4 |  |  | Inositol-5-monophosphate dehydrogenase (GuaB) | cytoplasmic membrane |
| A2 |  | ***Carbohydrate and metabolism*** | Triosephosphatase isomerase (TpiA) | unknown |
| E1 | CELLULAR PROCESSES | ***Cell envelope biogenesis*** | Glysosyl transferase (RfaG) | cytoplasmic |
| H3 |  |  | UDP-N-acetylglucosamine pyrophosphorylase (GlmU) | cytoplasmic |
| F6, G6, H6 |  | ***Post-translation modification and chaperones*** | Molecular chaperone (DnaK) | unknown |
| ***Commonly detected in mid-log and early-stationary phase culture supernatant*** | | | | |
| B4 | METABOLISM | ***Nucleotide transport and metabolism*** | Inositol-5-monophosphate dehydrogenase (GuaB) | cytoplasmic membrane |
| A5 |  |  | Glu/Leu/Phe/Val dehydrogenase (GdhA) | cytoplasmic |
| F2 |  | ***Carbohydrate and metabolism*** | Glyceraldehyde-3-phosphate dehydrogenase (GapA) | cytoplasmic |
| B7, C7 |  |  | Phosphopyruvate hydratase (Eno) | cytoplasmic |
| B2 |  |  | Propionyl-CoA carboxylase | cytoplasmic membrane |
| **^†^** B5 |  |  | 3-oxoaclyl-(acyl carrier protein) synthase II (FabB) | cytoplasmic |
| H5 |  |  | β-ketoacyl synthase | cytoplasmic |
| A6 |  |  | β-ketoadipyl CoA thiolase | cytoplasmic |
| G2 |  | ***Amino acid transport and metabolism*** | FAD dependent oxidoreductase (DadA) | cytoplasmic |
| B3 |  |  | Aspartate-semialdehyde dehydrogenase (Asd) | unknown |
| D3, H4 |  |  | Alanine dehydrogenase (AlaDH) | cytoplasmic |
| D5 |  |  | Acetylornithine deacetylase (ArgE) | unknown |
| C3 |  | ***Energy production and conversion*** | 3-methyl-2oxobutanoate dehydrogenase (AcoA) | cytoplasmic |
| C4 |  |  | Glycine dehydrogenase | unknown |
| E4 |  |  | Branched chain α-keto acid dehydrogenase (AceF) | cytoplasmic |
| F5 |  |  | Putative dihydrolipoamide dehydrogenase (Lpd) | cytoplasmic |
| E7 |  |  | Vanillate monooxgenase | unknown |
| **^†, #^** C9 |  |  | Phospholipid/glycerol acyltransferase | cytoplasmic membrane |
| E5 |  | ***Coenzyme metabolism*** | S-adenosylmethionine synthetase (MetK) | unknown (multiple) |
| **^†, #^** G3 | CELLULAR PROCESSES | ***Cell envelope biogenesis*** | Cell-wall associated hydrolase (Spr) | unknown (multiple) |
| G4 |  |  | D-Alanine-A-alanine ligase | cytoplasmic |
| **^†^** D4 |  | ***Cell motility and secretion*** | Flagellar hook-associated 2 domain protein (FliD) | extracellular |
| D6 |  | ***Inorganic ion transport and metabolism*** | Sulfatase (AslA) | unknown |
| C5 |  | ***Cell division*** | Cell division protein (FtsZ) | unknown (multiple) |
| G8 |  |  | Cell division protein (FtsA) | cytoplasmic |
| H1 |  | ***Post-translation modification and chaperones*** | Peroxiredoxin (AhpC) | cytoplasmic |
| **^†^** E3 |  |  | TonB-dependent siderophore (Fiu) | outer membrane |
| F4 |  |  | ATP-dependent protease ATP- binding | cytoplasmic |
| E6 |  |  | 60 kDa chaperonin (GroL) | cytoplasmic |
| B6 | INFORMATION STORAGE AND PROCESSING | ***Phage-related protein*** | Phage SP01 DNA polymerase | unknown |
| D7 |  |  | DEAD/DEAH box helicase | cytoplasmic |
| G1 |  | ***Translation*** | Ribosome recycling factor (Frr) | cytoplasmic |
| E2 |  |  | Elongation factor Ts (Tsf) | cytoplasmic |
| G5 |  |  | Asparty/glutamly-tRNA amidotransferase (GatB) | unknown |
| D9 |  |  | Tyrosyl-tRNA synthase | cytoplasmic |
| A3 | Poorly characterised | ***General function prediction*** | Zn-dependent alcohol dehydrogenase (AdhP) | cytoplasmic |
| ***Newly-released into early-stationary phase culture supernatant*** | | | | |
| F11 | METABOLISM | ***Nucleotide transport and metabolism*** | Adenylosuccinate synthetase (PutA) | unknown |
| C8 |  | ***Carbohydrate and metabolism*** | Pyruvate kinase (PykF) | unknown |
| F8, F9 |  |  | Transketolase (TktA) | unknown |
| E10 |  |  | Phosphoglycerate kinase (Pgk) | cytoplasmic |
| G10 |  | ***Lipid metabolism*** | 3-oxoacid CoA-transferase (AtoD) | cytoplasmic membrane |
| G12 |  |  | (3R)-hydroxymyristoyl-ACP dehydratase | unknown |
| E9 |  | ***Amino acid transport and metabolism*** | 2-aminoethylphosphonate-pyruvate transaminase | unknown |
| A12 |  |  | Aspartate ammonia-lyase (AspA) | cytoplasmic |
| A8 |  | ***Energy production and conversion*** | Pyruvate carboxylase | unknown |
| E11 |  |  | Aldehyde dehydrogenase | cytoplasmic |
| B12 |  |  | Delta-1-pyrroline-5- carboxylate dehydrogenase | cytoplasmic |
| F12 | CELLULAR PROCESSES | ***Cell envelope biogenesis*** | Glycosyl transferase (RfaG) | cytoplasmic |
| H12 |  |  | Capsule polysaccharide biosysnthesis | cytoplasmic |
| **^†, #^** B8 |  | ***Signal transduction mechanism*** | Integral membrane sensor signal histidine kinase (BaeS) | cytoplasmic membrane |
| **^†^** H9 |  | ***Cell motility and secretion*** | Flagellar hook-associated protein (FlgK) | unknown |
| **^†^** H11, C12, D12 |  |  | Flagellar hook-associated 2 domain protein (FliD) | extracellular |
| D10 |  | ***Inorganic ion transport and metabolism*** | Sulfatase (AslA) | unknown |
| C11, D11 |  |  | TonB-dependent siderophore (Fiu) | outer membrane |
| D8 | INFORMATION STORAGE AND PROCESSING | ***Translation*** | Polyribonucleotide nucleotidyltransferase (Pnp) | cytoplasmic |
| E8, A9 |  |  | Elongation factor G (FusA) | cytoplasmic |
| F10 |  |  | Methionine aminopeptidase (Map) | cytoplasmic |
| A11 |  |  | Peptidyl-tRNA hydrolase (Pth) | unknown |
| G9 |  | ***Transcription*** | DNA-directed RNA polymerase (RpoA) | cytoplasmic |
| B11 |  |  | ROK family protein (NagC) | cytoplasmic |
| H7 |  |  | Two-component transcriptional regulator | cytoplasmic |
| G7 | Poorly characterised | ***General function prediction*** | Hypothetical protein | cytoplasmic |
| A10, C10 |  |  | Hypothetical protein | unknown |
| H10 |  |  | Hypothetical protein | unknown |
| G11 |  |  | Conserved hypothetical protein (chitinase) | unknown |

**^a^** Functional category based on Clusters of Orthologous Groups (COG) of protein

**^b^** Subcellular location predicted using the PSORT analysis

**^†^** Presence of signal peptide predicted by SignalP server v. 3.0

**^#^**Number of predicted transmembrane helices in protein using TMHMM server v. 2.0
